# PAI1: a novel PP1‐interacting protein that mediates human plasma's anti‐apoptotic effect in endothelial cells

**DOI:** 10.1111/jcmm.13127

**Published:** 2017-03-11

**Authors:** Hui Yao, Guangchun He, Chao Chen, Shichao Yan, Lu Lu, Liujiang Song, K. Vinod Vijayan, Qinglong Li, Li Xiong, Xiongying Miao, Xiyun Deng

**Affiliations:** ^1^ Department of Pathology Hunan Normal University Medical College Changsha Hunan China; ^2^ Department of Pediatrics Hunan Normal University Medical College Changsha Hunan China; ^3^ Department of Medicine Baylor College of Medicine and Center for Translational Research on Inflammatory Diseases (CTRID) Michael E. DeBakey Veterans Affairs Medical Center (MEDVAMC) Houston TX USA; ^4^ Department of Surgery University of Pittsburgh Medical Center Pittsburgh PA USA; ^5^ Department of General Surgery Second Xiangya Hospital Central South University Changsha Hunan China

**Keywords:** plasminogen activator inhibitor 1, protein phosphatase 1, PP1‐interacting protein, apoptosis, endothelial cells

## Abstract

Activation of apoptotic signalling in endothelial cells contributes to the detrimental effects of a variety of pathological stimuli. In investigating the molecular events underlying the anti‐apoptotic effect of human plasma in cultured human endothelial cells, we unexpectedly uncovered a novel mechanism of apoptosis suppression by human plasma through an interaction between two previously unrelated proteins. Human plasma inhibited hypoxia–serum deprivation‐induced apoptosis and stimulated BAD^S136^ and Akt^S473^ phosphorylation. Akt1 silencing reversed part (~52%) of the anti‐apoptotic effect of human plasma, suggesting the existence of additional mechanisms mediating the anti‐apoptotic effect other than Akt signalling. Human plasma disrupted the interaction of BAD with protein phosphatase 1 (PP1). Mass spectrometry identified fourteen PP1‐interacting proteins induced by human plasma. Notably, a group of serine protease inhibitors including plasminogen activator inhibitor 1 (PAI1), a major inhibitor of fibrinolysis, were involved. Silencing of PAI1 attenuated the anti‐apoptotic effect of human plasma. Furthermore, combined Akt1 and PAI1 silencing attenuated the majority of the anti‐apoptotic effect of human plasma. We conclude that human plasma protects against endothelial cell apoptosis through sustained BAD phosphorylation, which is achieved by, at least in part, a novel interaction between PP1 with PAI1.

## Introduction

Normal organ function relies upon the maintenance of vascular homeostasis and the integrity of the endothelial lining of blood vessels. Endothelial cell (EC) survival/apoptosis plays important roles in the homeostasis of the vascular system. EC apoptosis is known to alter cell morphology by interrupting the cell–cell and cell–matrix interaction, resulting in eventual removal of ECs from their underlying basement membrane. Therefore, anomalous EC apoptosis is considered a critical step that provokes acute endothelial dysfunction and plays an important role in the development of numerous disease conditions such as atherosclerosis and chronic transplant vasculopathy 1, 2. Our body system possesses a well‐developed mechanism of maintaining vascular homeostasis through the prevention of EC injury caused by abnormally increased apoptosis.

Members of the Bcl‐2 family are key regulators of apoptosis that include both anti‐apoptotic and pro‐apoptotic proteins 3. The pro‐apoptotic BH3‐only protein BAD plays a critical role in the regulation of EC apoptosis *in vivo* and *in vitro* 4. In its unphosphorylated form, BAD triggers the release of mitochondrial apoptogenic factors into the cytoplasm, leading to loss of mitochondrial functions and subsequent caspase activation. Phosphorylation of BAD by protein kinases such as PKB/Akt and PKA results in binding of BAD to 14‐3‐3 proteins 5. The binding of BAD to 14‐3‐3 displaces BAD from complexing with its anti‐apoptotic partners, such as Bcl‐xL, and blocks the ability of BAD to induce cell death, thus promoting cell survival 6. On the contrary, dephosphorylation of BAD by protein phosphatases (PPs) results in dissociation of BAD from 14‐3‐3, translocation to the mitochondria and subsequent association with the anti‐apoptotic protein Bcl‐xL, thus promoting apoptosis 5.

In this study, we investigated the anti‐apoptotic effect of human plasma (HP) and the underlying molecular mechanisms using an *in vitro* model of EC injury. We found that HP suppressed EC apoptosis, at least partly, *via* the stimulation of phosphorylation of the pro‐apoptotic protein BAD. Through liquid chromatography–tandem mass spectrometry (LC‐MS/MS), co‐immunoprecipitation and glutathione *S*‐transferase (GST) pull‐down assay, we identified PAI1 as a novel PP1‐interacting protein (PIPs), which plays an important role in HP‐mediated BAD phosphorylation and anti‐apoptotic effect in ECs.

## Materials and methods

### Reagents and antibodies

Antibodies against phospho‐Akt (Ser473) and phospho‐BAD (Ser136) were purchased from Cell Signaling Technology (Danvers, MA, USA). Antibodies against BAD, 14‐3‐3, Akt, PP1 (recognizing all isoforms), GAPDH and an isotype‐matched IgG control for immunoprecipitation were purchased from Santa Cruz Biotechnology (Santa Cruz, CA, USA). Anti‐PAI1 antibody was purchased from BD Transduction Laboratories (Sparks, MD, USA). The siRNAs against Akt1, PAI1 (SERPIN E1) and the non‐targeting mock siRNA were purchased from Dharmacon (Waltham, MA, USA). Lipofectamine 2000, Dynabeads Protein G and foetal bovine serum (FBS) were obtained from Life Technologies (Carlsbad, CA, USA). HP was obtained by routine plasma separation technique under aseptic conditions from healthy donors.

### Cell culture and experimental treatments

Human pulmonary microvascular ECs (HPMECs) and human cardiac microvascular ECs (HCMECs) were purchased from PromoCell (Heidelberg, Germany). These cells were routinely cultured in EC growth medium MV2 containing 10% FBS supplemented with the respective growth supplement pack as recommended by the manufacturer. Both cell types were tested negative for mycoplasma and were used within passage 10 for all experiments. For normoxic culture, the cells were incubated in a 37°C incubator with 21% O_2_ and 5% CO_2_. Hypoxia (1% O_2_ and 5% CO_2_) was achieved in a hypoxia chamber (Thermo Fisher Scientific, Waltham, MA, USA) through injecting N_2_ gas.

**Figure 1:**
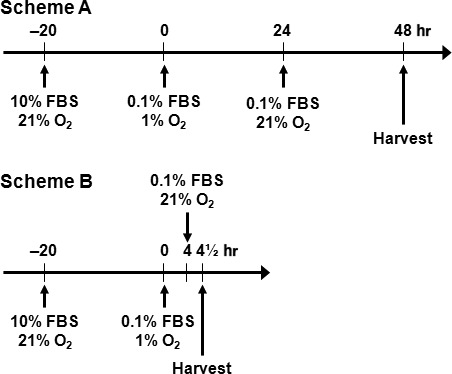
The HR/SS cell treatment protocols. Two protocols of hypoxia–reoxygenation (HR) and serum starvation (SS) were employed for the treatment of cells. Scheme A, after seeding, the cells were cultured in starvation medium (containing 0.1% FBS) under hypoxia (1% O_2_) for 24 hrs followed by treatment with HP for 24 hrs under normoxia (21% O_2_). Scheme B, after seeding, the cells were cultured in starvation medium (containing 0.1% FBS) under hypoxia (1% O_2_) for 4 hrs followed by treatment with HP for 30 min. under normoxia (21% O_2_). Scheme A and Scheme B are used for cell apoptosis and signalling studies, respectively.

Two protocols of hypoxia–reoxygenation (HR) 7, 8 and serum starvation (SS) of cell treatment were employed. For apoptosis analysis, the cells seeded in 96‐well plates (1.2 × 10^4^ cells/well) were allowed to adhere overnight before the HR/SS procedure. To induce apoptosis, the cells were cultured in starvation medium containing 0.1% FBS under hypoxia (1% O_2_) for 24 hrs followed by treatment with various concentrations of HP for another 24 hrs under normoxia (21% O_2_) (Fig. 1 Scheme A). For all other experiments, the cells seeded in 6‐cm dishes (2.5 × 10^5^ cells/dish) were grown overnight in regular culture medium containing 10% FBS under normoxia. To induce signalling changes, the cells were changed to medium containing 0.1% FBS and starved under hypoxia for 4 hrs. HP was added at the end of starvation and the treatment continued for 30 min. under hypoxia (Fig. 1 Scheme B). For both schemes, to keep the total volume constant after HP addition, the corresponding volume of the culture medium was removed before the same amount of HP added to each treatment group.

### Apoptosis analysis

To induce apoptosis, we used a HR/SS protocol as described in Figure 1 Scheme A. After culture in regular culture conditions for 24 hrs, the cells were serum‐starved with medium containing 0.1% FBS under hypoxia for 24 hrs (1% O_2_). HP was added at the beginning of reoxygenation (21% O_2_). At the end of the experimental procedure, the same volume (50 μl from each well) of conditioned media was collected and used for caspase‐3/7 activity assay using a luminescent Caspase‐Glo 3/7 Assay kit (Promega, Madison, WI, USA) according to the manufacturer's instructions. At the same time, the cells were lysed and used for the detection of fragmented nucleosomes using a Cell Death Detection kit (Roche, Indianapolis, IN, USA) as described 9.

### siRNA‐mediated gene silencing

ECs (1.2 × 10^5^ cells/6‐cm dish) were transfected with siRNA against Aktl, PAI1 or a scramble mock siRNA at a final concentration of 100 nM using Lipofectamine 2000 as recommended by the manufacturer. Seventy‐two hours after transfection, the cells were subjected to the HR/SS procedure and treated with HP followed by whole‐cell lysate preparation and immunoblotting according to the procedure described in Figure 1 Scheme B.

### Co‐immunoprecipitation, gel staining and immunoblotting

Whole‐cell lysates were prepared from cultured cells using 1 × cell lysis buffer (Cell Signaling Technology) with 1 × protease inhibitor cocktail (Complete Mini, Roche) and 1 mM PMSF (Sigma‐Aldrich, St Louis, MO, USA) added. For co‐immunoprecipitation, 100 μg of whole‐cell lysate was immunoprecipitated with 0.2 μg of an antibody against BAD, PP1 or isotype‐matched control IgG for 1 hr at 4°C followed by overnight incubation with 20 μl of washed Dynabeads Protein G (Life Technologies) at 4°C. Bound proteins were washed three times with PBS containing 0.02% Tween‐20 and eluted with 1.5 × sample buffer containing 50 mM DTT (Life Technologies). After boiling, an equal volume of each immunoprecipitation sample or whole‐cell lysate was separated on a 4–12% gradient density NuPAGE Novex Bis–Tris precast denaturing gel (Life Technologies). The separated proteins were subjected to either silver staining or immunoblotting and ECL development according to our standard protocol 10. For quantification of immunoblot bands, the grey scale densities were obtained using the LI‐COR Image Studio Digits Software V3.1 (Lincoln, NE, USA) and the relative intensities calculated by normalizing against the loading control from at least two independent experiments.

### MS analysis and database searching

Gel bands from control and treated cells were excised, and in‐gel digestion was performed. The tryptic digests were taken to dryness in a Thermo SpeedVac and dissolved in 20 μl of 2% acetonitrile and 0.1% formic acid in water (Solvent A). Samples were analysed on a LTQ Orbitrap XL (Thermo Fisher Scientific) interfaced with an Eksigent nano‐LC 2D plus ChipLC system (Eksigent Technologies, Dublin, CA, USA). Reversed‐phase C18 chromatographic separation of peptides was carried out on a ChromXP C18‐CL column (75 μm i.d. × 10 cm length, 3 μm) at 300 nl/min., with column temperature controlled at 60°C. Gradient conditions were as follows: 3–8% Solvent B (0.1% formic acid in acetonitrile) for 5 min.; 8–33% Solvent B for 120 min.; 33–90% Solvent B for 10 min.; 90% Solvent B held for 10 min.; 90–3% for 5 min. The total run time was 150 min. Data analysis was performed with MaxQuant software, supported by Mascot (version 3.2.02) as a database search engine for peptide identification using following criteria: database: human SwissProt; enzyme: trypsin; miscleavage: 2; MS tolerance: 10 ppm; MS/MS tolerance: 0.8 Da; fixed modifications: carbamidomethylation; variable modifications: oxidation (M).

### Construction of PP1‐GST fusion proteins and GST pull‐down

Four PP1 isoforms, *that is* PP1α, PP1β, PP1γ1 and PP1γ2, the latter two arising from the same gene through alternative splicing, have been identified with near 90% amino acid sequence identity 11. The cDNAs for PP1α, PP1β and PP1γ1 in pCMV vector were amplified by PCR and subcloned into a GST vector pGEX 4T‐1 (GE Heathcare Biosciences, Piscataway, NJ, USA). GST‐tagged PP1α, PP1β, PP1γ1 proteins and control GST protein were expressed in *E*. *Coli* following induction with isopropyl‐β‐D‐thiogalactopyranoside (IPTG) and purified using glutathione–sepharose beads (GE Healthcare, Pittsburgh, PA, USA) 12. Standard GST pull‐down assay was performed as previously described 13. After pull‐down, the proteins were eluted with 1.5 × sample buffer containing 50 mM DTT and resolved by 4–12% Bis‐Tris denaturing gel electrophoresis followed by immunoblotting as described above.

### Statistical analysis

All quantitative values were presented as mean ± S.D. from at least two independent experiments. One‐way anova was used for multiple group comparisons of means followed by the Holm–Sidak *post hoc* test using SigmaPlot 12.5. *P* < 0.05 was considered statistically significant.

## Results

### Human plasma protects against EC apoptosis

**Figure 2:**
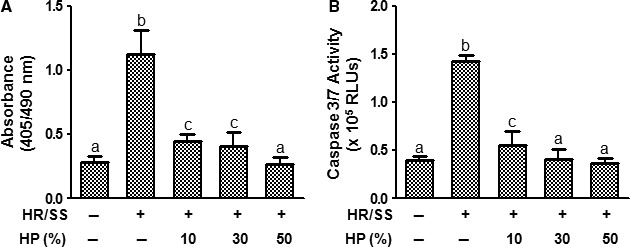
Human plasma protects endothelial cells against hypoxia–reoxygenation and serum starvation‐induced apoptosis. HPMECs were cultured and treated for 24 hrs with various concentrations of HP according to the procedure described in Scheme A. The HR/SS (−) control cells were cultured under normoxia and without serum starvation during the whole procedure. At the end of the experiment, the cells were lysed and subjected to detection of DNA fragmentation using the Cell Death Detection kit (**A**). Alternatively, conditioned media were collected at the end of the experiment and subjected to caspase 3/7 activity analysis using the Caspase‐Glo 3/7 Assay kit (**B**). Values were mean ± S.E.M. from three independent experiments. Different symbols indicate *P* < 0.05 between the groups; identical symbols indicate no significant difference. Note: Different lower‐case letters denote significant statistical difference (*P* < 0.05) among different groups. HR: hypoxia–reoxygenation; SS: serum starvation; RLUs: relative light units.

To determine whether HP has a protective effect against EC apoptosis under a culture condition that induces cell injury, we used an *in vitro* EC model of HR/SS (Fig. 1 Scheme A) to induce EC apoptosis. The apoptosis induced by HR/SS was indeed inhibited by HP as demonstrated by dose‐dependent inhibition of DNA fragmentation (Fig. 2A) and caspase 3/7 activity (Fig. 2B) in HPMECs.

### Human plasma maintains BAD phosphorylation by activating Akt and preventing the interaction of PP1 with BAD

**Figure 3:**
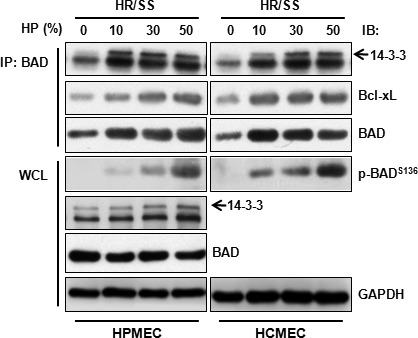
Human plasma induces phosphorylation of BAD and increases its interaction with 14‐3‐3 in endothelial cells. ECs were treated for 30 min. with HP at the indicated concentrations according to the procedure described in Scheme B and subjected to whole‐cell lysate preparation followed by immunoblotting or immunoprecipitation–immunoblotting. IP: immunoprecipitation; IB: immunoblotting; WCL: whole‐cell lysate.

Given the fact that the maintenance of BAD phosphorylation plays a central role in the inhibition of apoptosis in ECs *in vivo* and *in vitro* 4, we asked whether inhibition of EC apoptosis by HP was related to the change of the phosphorylation status of BAD. As expected, when used at concentrations ranging from 10% to 50%, HP indeed induced dose‐dependent phosphorylation of BAD at Ser136 in both HPMECs and HCMECs (Fig. 3). Phosphorylation of BAD was also up‐regulated by HP at another critical site, Ser112 (data not shown). In support of the anti‐apoptotic role of phosphorylated BAD, HP increased interaction of BAD with 14‐3‐3 proteins in both cell types. Increased interaction between BAD and 14‐3‐3 is supposed to result in decreased binding of BAD to Bcl‐xL 6. We examined this possibility in the case of HP treatment in ECs. However, for unknown reasons, we did not observe a decrease in the binding of BAD to Bcl‐xL upon HP treatment in both EC types (Fig. 3).

**Figure 4:**
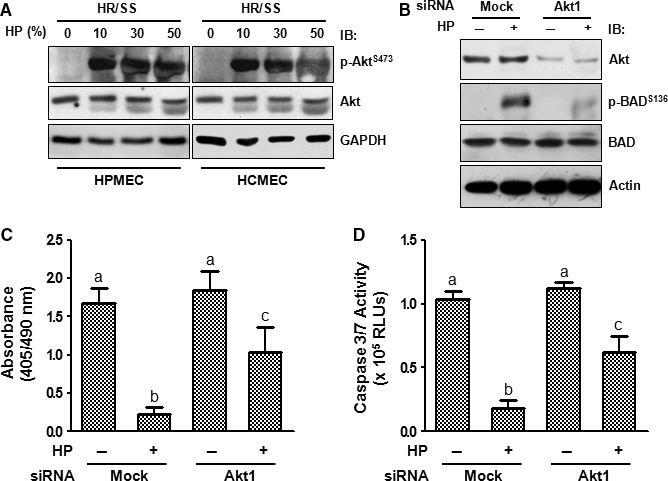
Akt is partially responsible for the anti‐apoptotic effect of human plasma. ECs were treated according to Scheme A (4**C** and 4**D**) or Scheme B (4**A** and 4**B**). (**A**) ECs treated with different concentrations of HP were subjected to whole‐cell lysate preparation and immunoblotting. (**B**–**D**), HPMECs were transfected with Akt1 siRNA or a scramble non‐targeting control siRNA using Lipofectamine 2000. Seventy‐two hours after transfection, the cells were starved and treated for 30 min. with or without HP (30%) followed by immunoblotting (**B**), or subjected to DNA fragmentation (**C**), or caspase 3/7 activity assay (**D**). Note: Different lower‐case letters denote significant statistical difference (*P* < 0.05) among different groups. IB: immunoblotting; RLUs: relative light units.

Next, we investigated the upstream kinases that are involved in HP‐mediated BAD phosphorylation. Two major kinases, PKB/Akt and PKA, are involved in multiple‐site phosphorylation of BAD 5. As depicted in Figure 4A, we observed a dose‐dependent increase in phosphorylation of Akt at Ser473 in both HPMECs and HCMECs. HP also up‐regulated the phosphorylation of the catalytic subunit of PKA (Thr197) but at a much lower level (data not shown), suggesting that Akt plays a much more important role in HP‐induced BAD phosphorylation.

The role of Akt in HP‐mediated BAD phosphorylation and apoptosis inhibition was investigated by genetic manipulation. We knocked down Akt1, which is the major Akt isoform in ECs 14, by siRNA‐mediated gene silencing. As shown in Figure 4B, knockdown of Akt1 resulted in a decrease in Akt level by 79.1 ± 2.0% and a decrease in HP‐induced BAD phosphorylation at Ser136 (62.3 ± 4.6%) in HPMECs. As expected, Akt1 silencing also reversed HP's inhibition of DNA fragmentation (51.8 ± 8.1%) (Fig. 4C) and caspase 3/7 activity (53.5 ± 4.0%) (Fig. 4D).

**Figure 5:**
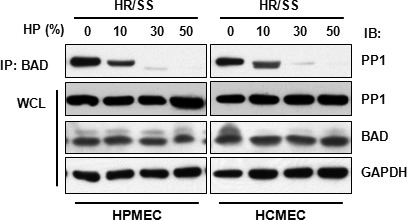
Human plasma decreases the interaction of PP1 with BAD in endothelial cells. ECs treated with different concentrations of HP according to Scheme B were subjected to immunoprecipitation–immunoblotting. IP: immunoprecipitation; IB: immunoblotting; WCL: whole‐cell lysate.

As Akt silencing only partially reversed HP's anti‐apoptotic effect (Fig. 4C and D), the participation of other anti‐apoptotic mechanisms was also investigated. PPs, specifically PP1 and PP2B, dephosphorylate BAD at Ser136 and switch BAD from being anti‐apoptotic to pro‐apoptotic 5. As PP1 catalyses the majority of protein dephosphorylation reactions in eukaryotic cells 15, we tested whether PP1 is also involved in HP‐mediated BAD phosphorylation in the scenario of EC injury. HP dose‐dependently decreased the interaction of PP1 with BAD in both HPMECs and HCMECs (Fig. 5). Dissociation of PP1 from BAD prevents the dephosphorylation of BAD by PP1, thereby maintaining BAD in a hyperphosphorylated anti‐apoptotic state in conjugation with HP‐induced Akt activation.

### Identification of PAI1 as a PP1‐interacting protein

**Figure 6:**
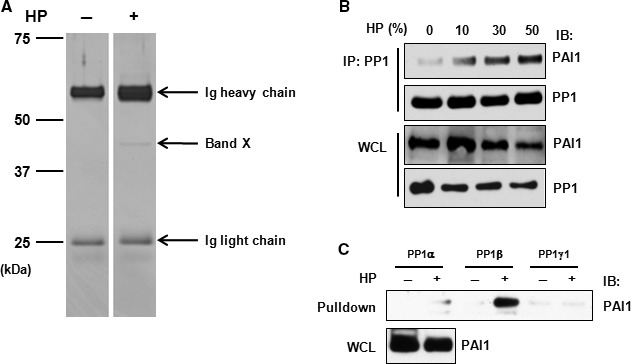
Identification of PAI1 as a PP1‐interacting protein. (**A**) HCMECs were treated with or without HP for 30 min. according to Scheme B and whole‐cell lysates subjected to immunoprecipitation using an anti‐PP1 antibody followed by SDS‐PAGE separation and silver staining. The positions of molecular mass markers are marked on the left. Band X is the gel band of interest which was subjected to LC‐MS/MS analysis. (**B**) HCMECs were treated with HP for 30 min. and whole‐cell lysates were prepared and subjected to immunoprecipitation–immunoblotting. (**C**) Whole‐cell lysates prepared from HCMECs treated with or without HP were incubated with an equal molar amount of GST‐tagged PP1 isoforms followed by pull‐down with glutathione‐sepharose beads. The eluted proteins were resolved by SDS‐PAGE followed by immunoblotting. Ig, immunoglobulin; IP, immunoprecipitation; IB: immunoblotting; WCL: whole‐cell lysate.

In search for the novel anti‐apoptotic mechanisms involved in EC protection, we employed LC‐MS/MS, co‐immunoprecipitation and GST pull‐down assays in an attempt to identify PIPs that are important for HP's protective effects in ECs. HCMECs were treated with or without HP and subjected to immunoprecipitation with an anti‐PP1 antibody. The proteins that were pulled down with the anti‐PP1 antibody were separated by SDS‐PAGE and visualized by silver staining. One PP1‐interacting band around 37–50 kD (Band X) was consistently present in HP‐treated HCMECs in repeated experiments (Fig. 6A). This Band X was excised and subjected to tryptic digestion and LC‐MS/MS. Through database search, 14 PIPs induced by HP were identified (Table 1). Of particular interest were a group of serine protease inhibitors, such as PAI1, serine protease inhibitor 2 and serine protease inhibitor B9, demonstrating the importance of this group of proteins in the interaction with PP1.

**Table 1:** Identification of HP‐induced PP1‐interacting proteins in endothelial cells

No	E‐value	Protein name	Protein description	Protein function	Accession Number
1	2.20E‐05	PAI1	Plasminogen activator inhibitor 1 (serine protease inhibitor E1)	Serine protease inhibitor	P05121
2	2.50E‐05	DJB11	DnaJ homolog subfamily B member 11	Heat‐shock protein binding	Q9UBS4
3	2.20E‐05	SPI2	Serine protease inhibitor 2	Serine protease inhibitor	P33830
4	1.70E‐03	ANM2	Protein arginine N‐methyltransferase 2	Arginine methyltransferase	P55345
5	1.90E‐07	ILF2	Interleukin enhancer‐binding factor 2	Transcriptional activator	Q12905
6	4.90E‐04	GNAT2	Guanine nucleotide‐binding protein G(t) subunit alpha‐2	Guanine nucleotide‐binding proteins (G proteins)	P19087
7	2.50E‐03	SERPINB9	Serine protease inhibitor B9	Serine protease inhibitor	P50453
8	8.50E‐04	ADRM1	Proteasomal ubiquitin receptor ADRM1	Proteasomal ubiquitin receptor	Q16186
9	1.60E‐03	S1PR5	Sphingosine 1‐phosphate receptor 5	Receptor for the lysosphingolipid sphingosine 1‐phosphate (S1P)	Q9H228
10	5.50E‐04	SIAS	Sialic acid synthase	N‐acetylneuraminate biosynthesis	Q9NR45
11	1.90E‐02	K1C19	Keratin, type I cytoskeletal 19	Organization of myofibers	P08727
12	8.30E‐03	ADRB3	Beta‐3 adrenergic receptor, isoform CRA_b	G protein‐coupled receptor	A8KAG8
13	2.50E‐03	PGK1	Phosphoglycerate kinase 1	Gluconeogenesis; glycolysis	P00558
14	2.10E‐02	ADAM5	Putative disintegrin and metalloproteinase domain‐containing protein 5	Metalloendopeptidase activity	Q6NVV9

Serine protease inhibitor 2 has previously been reported to be one of the PIPs in vertebrates 16. PAI1 (molecular weight of about 45–48 kD) is a fibrinolysis‐regulatory protein with known apoptosis‐regulatory activity in ECs 17. To our knowledge, the interaction between PAI1 and PP1 has not been previously reported. We thus investigated whether PAI1 is a novel intracellular PIP that protects against apoptosis in ECs. The interaction between PP1 and PAI1 was confirmed by co‐immunoprecipitation followed by immunoblotting in HCMECs (Fig. 6B). Unexpectedly, we found that treatment with HP, especially at a high concentration (50%), led to a decrease in total PAI1 protein level. The reason for this is not known and might be an interesting issue for further investigation.

Four isoforms of PP1 (PP1α, β, γ1 and γ2) have been identified in mammals thus far 11. To further confirm the PP1‐PAI1 interaction and ascertain the specific isoform of PP1 engaged in PAI1 binding, we performed GST pull‐down assay using purified PP1‐GST fusion proteins. The interaction of PAI1 was prominently noticed with PP1β (>>PP1α >PP1γ1) in HP‐treated HCMECs (Fig. 6C). Previous studies by others have demonstrated that it is the PP1α, not PP1β, isoform that can be targeted to BAD by the Bcl‐2 family proteins 18, 19, 20. The difference between our findings and these reports might be because of the prevalence of different PP1 isoforms in different tissue types involved. The significance of this isoform‐specific preference of PP1 interaction with PAI1 is not clear yet.

### Gene silencing of PAI1 suppresses BAD phosphorylation and promotes EC apoptosis

**Figure 7:**
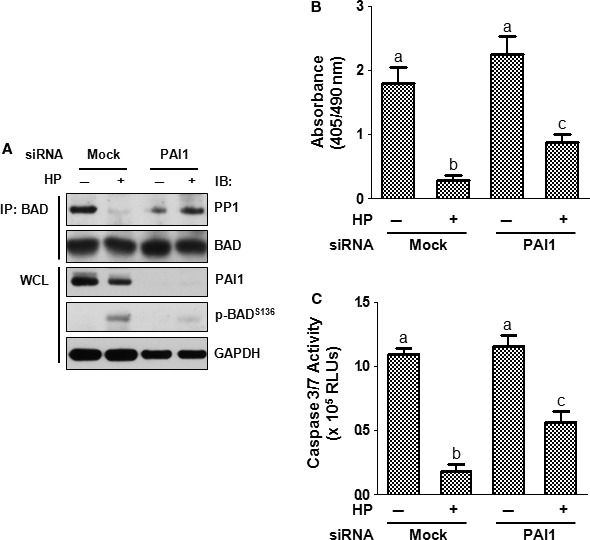
PAI1 is responsible for part of human plasma‐mediated BAD phosphorylation and apoptosis protection. ECs were transfected with PAI1 siRNA using Lipofectamine 2000 and subjected to immunoprecipitation–immunoblotting (**A**), DNA fragmentation (**B**) or caspase 3/7 activity assay (**C**). Note: Different lower‐case letters denote significant statistical difference (*P* < 0.05) among different groups. IB, immunoblotting; WCL, whole‐cell lysate; RLUs, relative light units.

Based on the above observations of the PP1‐PAI1 interaction, we ascertained whether this interaction has a pathophysiological role in maintaining the anti‐apoptotic signals. To this end, HCMECs were silenced for PAI1 by siRNA and analysed for DNA fragmentation and caspase 3/7 activity upon HP treatment. Knockdown of PAI1 by siRNA resulted in near complete elimination of total PAI1 and attenuated HP‐induced BAD^S136^ phosphorylation by 82.7 ± 3.5% (Fig. 7A). Furthermore, PAI1 silencing reversed HP's inhibition of DNA fragmentation by 43.0 ± 1.5% and caspase 3/7 activity by 39.4 ± 2.9% (Fig. 7B and C). These data suggest that HP induces BAD phosphorylation and promotes EC survival through promoting the interaction between PAI1 and PP1 and displacing PP1 from complexing with BAD.

## Discussion

**Figure 8:**
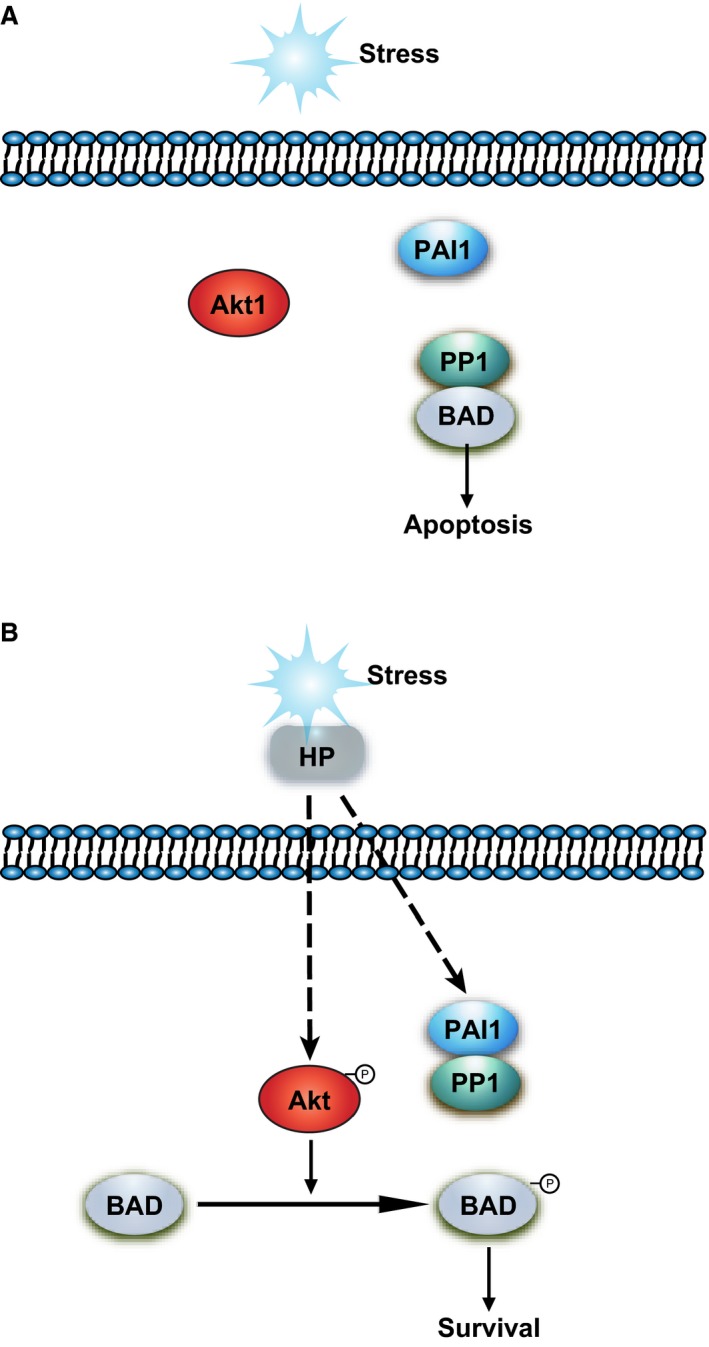
Schematic representation of the role of PAI1‐PP1 interaction in the regulation of BAD phosphorylation and the anti‐apoptotic effect in endothelial cells. (**A**) Under stress conditions such as hypoxia–reoxygenation and serum deprivation, PP1 binds to BAD, rendering BAD in an unphosphorylated state and the cell eventually undergoes apoptosis. (**B**) HP induces BAD phosphorylation and promotes EC survival through promoting the interaction between PAI1 and PP1 and displacing PP1 from complexing with BAD. Activation of Akt signalling also contributes to the phosphorylation of BAD in this process.

Increased EC apoptosis leads to the disruption of vascular integrity, contributing to the pathogenesis of a variety of pathological conditions, such as ischaemia–reperfusion injury. As humans, we have developed a delicate system to maintain vascular stability through the prevention or repair of EC apoptosis. In the present study, using a model of HR combined with serum deprivation (SS), we have shown that HP significantly stimulated phosphorylation of BAD and inhibited HR/SS‐induced vascular EC apoptosis. The underlying signalling mechanisms involved elevation of Akt‐mediated BAD phosphorylation in conjugation with displacement of PP1 from BAD, thereby preventing BAD dephosphorylation. More importantly, we have identified PAI1 as a novel PIP which is involved in HP‐mediated anti‐apoptosis in ECs. Together, activation of these two interdependent pathways results in elevated BAD phosphorylation and is responsible for HP's anti‐apoptotic effect in ECs. A summary of the signalling pathways mediating HP's regulation of BAD phosphorylation and anti‐apoptotic effect in ECs is depicted in Figure 8.

The phosphorylation status of BAD is a crucial regulator of cell survival and death. Two types of signals regulate the phosphorylation level of BAD, thus determining whether BAD serves as an anti‐apoptotic or pro‐apoptotic protein. While the Akt‐mediated pathway up‐regulates BAD phosphorylation, the PAI1‐regulated PP1‐dependent pathway down‐regulates it. In our study, we have demonstrated the importance of these two regulatory arms on BAD phosphorylation and cell survival through gene silencing experiments (Figs 4B and 7A). Nevertheless, we observed some residual anti‐apoptotic activity when either Akt1 or PAI was knocked down. This could be due to the residual protein levels, especially in the case of Akt1 knock‐down, and the subsequent BAD phosphorylation. In addition, the plasma is known to contain thousands of proteins covering a myriad of physiological and pathological functions 21, 22. Therefore, the possibility of the involvement of other factors such as IL‐2 20 or IL‐3 23 that promote BAD phosphorylation could not be excluded. Alternatively, other survival signals in the plasma may activate, for example, the MAPK‐MEK 24 or STAT3 25 signalling pathway and promote cell survival even in the absence of BAD phosphorylation.

PP1 belongs to the phosphoprotein phosphatase (PPP) superfamily of protein Ser/Thr phosphatases. The PP1 holoenzyme is composed of a catalytic subunit (commonly known as PP1) with various kinds of regulatory (or targeting) subunits. While the catalytic PP1 subunit determines the activity of the PP1 holoenzyme, the regulatory subunit determines substrate specificity and subcellular localization of PP1 26. Identification of the so‐called PIPs, the regulatory subunits of PP1, has been a challenging and exciting area of mammalian cell biology and molecular pharmacology 26, 27. Being much more tissue or subcellular compartment specific than the PPs, the PIPs provide better targets of therapeutic potential in cardiovascular diseases and cancer, thus limiting unnecessary toxic side effect and increasing specificity 11.

PAI1 is a single‐chain glycoprotein that belongs to the superfamily of SERPINs. PAI1 is the primary and most specific fast‐acting inhibitor of both the tissue‐type plasminogen activator (t‐PA) and the urinary‐type plasminogen activator (u‐PA) 17. PAI1 is involved in the so‐called vascular remodelling through complex regulation of a delicate balance between proliferation and apoptosis of vascular ECs and smooth muscle cells 28. With regard to apoptosis regulation, PAI1 can context‐dependently inhibit or promote cell apoptosis in ECs and smooth muscle cells 29, 30. Induced expression of exogenous PAI1 has been shown to inhibit prostate cancer growth though induction of tumour endothelial apoptosis, thus limiting angiogenesis 31. Furthermore, PAI1 protects ECs from Fas/FasL‐mediated apoptosis, establishing PAI1 as a potential target for anti‐angiogenic and antivascular therapies 32. These studies, together with ours, highlight the importance of PAI1 in vascular biology that in a broader sense involves cardiovascular complications and cancer development.

In summary, our study reveals a novel mechanism by which HP protects ECs from HR/SS‐induced apoptosis. PAI1, through its interaction with PP1 and together with Akt signalling, renders BAD in a hyperphosphorylated state, which mediates the anti‐apoptotic effect of HP in ECs. Identification of PAI1 as a novel PIP offers PAI1 a totally different mode of action, *that is*, through intracellular interaction with PP1 and regulation of BAD phosphorylation in ECs. The exact mechanisms by which PAI1 interacts with PP1 and how PAI1‐regulated BAD phosphorylation impacts cellular functions other than apoptosis need to be further investigated. The PP1‐docking motif in PAI1, similar to the one found in most other PIPs (*e.g*. RVxF) 15 but is absent in PAI1, needs to be identified. In addition, the specific factors in the plasma that are responsible for triggering PAI1‐PP1 interaction need to be further identified. In a broader context, identification of PAI1 as a novel PIP will open up a new avenue for intracellular signalling study in vascular biology.

## Conflict of interest statement

The authors confirm that there is no conflict of interest.
